# RheoStim: Development of an Adaptive Multi-Sensor to Prevent Venous Stasis

**DOI:** 10.3390/s16040428

**Published:** 2016-03-24

**Authors:** Sören Weyer, Fabio Weishaupt, Christian Kleeberg, Steffen Leonhardt, Daniel Teichmann

**Affiliations:** 1 Chair for Medical Information Technology, Helmholtz Institute, RWTH Aachen University, Pauwelsstr. 20, Aachen 52074, Germany; fabio.weishaupt@rwth-aachen.de (F.W.); leonhardt@hia.rwth-aachen.de (S.L.); teichmann@hia.rwth-aachen.de (D.T.); 2 tic Medizintechnik GmbH & Co. KG, Endelner Feld 9, Dorsten 46286, Germany; kleeberg@ticmed.de

**Keywords:** impedance plethysmography, reflective photoplethysmography, sensor data fusion, chronic venous insufficiency, Kalman filter, muscle pump electro-stimulation

## Abstract

Chronic venous insufficiency of the lower limbs is often underestimated and, in the absence of therapy, results in increasingly severe complications, including therapy-resistant tissue defects. Therefore, early diagnosis and adequate therapy is of particular importance. External counter pulsation (ECP) therapy is a method used to assist the venous system. The main principle of ECP is to squeeze the inner leg vessels by muscle contractions, which are evoked by functional electrical stimulation. A new adaptive trigger method is proposed, which improves and supplements the current therapeutic options by means of pulse synchronous electro-stimulation of the muscle pump. For this purpose, blood flow is determined by multi-sensor plethysmography. The hardware design and signal processing of this novel multi-sensor plethysmography device are introduced. The merged signal is used to determine the phase of the cardiac cycle, to ensure stimulation of the muscle pump during the filling phase of the heart. The pulse detection of the system is validated against a gold standard and provides a sensitivity of 98% and a false-negative rate of 2% after physical exertion. Furthermore, flow enhancement of the system has been validated by duplex ultrasonography. The results show a highly increased blood flow in the popliteal vein at the knee.

## 1. Introduction

Deformation of a leg vein is a common phenomenon. For example, whereas only about 10% of the German population is free of symptoms, 59% suffer (at least) from telangiectasia or similar syndromes [1]; the remaining 31% show severe symptoms due to chronic vein problems, such as varicose veins, fluid accumulation or venous leg ulcers.

A common treatment for venous stasis is compression therapy. The compression is usually induced by compression stockings, by which pressure is applied to the deep veins in the legs. The compliance of the compression stockings is considered negative, since patients complain of sweating, itching, cosmetic concerns, edema exacerbation, exudation lesions of the lower legs and application difficulty [2].

The method of external counter pulsation (ECP) is used to initiate a diastolic pulse wave in the arterial system by squeezing the inner leg vessels. The muscle pump compresses superficial and lower veins, as well as lymphatic vessels in the leg. This pumping mechanism increases venous blood flow from the legs back to the heart. In most studies, functional electrical stimulation (FES) was used to activate the muscle pump [3,4,5]. Alternatively, pneumatic leg cuffs were used, which compress the leg muscles by inflation of the cuffs in order to squeeze the inner arteries [6,7]. The beginning and duration of compression is either fixed or is estimated from the electrocardiogram (ECG) signal. It is not possible to measure the beginning of the diastolic phase from the ECG. Normally, it is realized by an R-wave detection algorithm, where the trigger point is set at a delay time after the occurrence of the R-wave. The delay time is the estimated period between R-wave and the end of the T-wave where the aortic valve closes. The R-wave-based triggers usually fail for patients with left bundle branch block or pacemaker spikes in the electrocardiogram [8]. Furthermore, the ECG-based trigger method needs three electrodes, which are placed on the chest.

Here, we introduce a new triggering method for stimulation of the muscle based on multi-sensor plethysmography and describe the hardware design and signal processing. The device is based on impedance plethysmography (IPG) and reflective photoplethysmography (rPPG) to detect the inflow of blood into the leg compartment. This allows one to determine the waveform of the blood flow over time by measurement of the pulsatile change of electrical impedance and light absorption in the observed segment. The appearance of the PPG and IPG pulse is commonly divided into two phases: the anacrotic phase is the rising edge of the pulse, whereas the catacrotic phase is the falling edge of the pulse. The first phase is primarily concerned with systole, and the second phase with diastole and wave reflections from the periphery [9]. Therefore, the merged signal is used to determine different phases of the cardiac cycle.

This information is then used to stimulate the muscle pump during the filling phase of the heart. In this work, the peak of the signal is used to trigger the stimulation, which is in the systolic phase. Crochetiere *et al*. investigated the temporal torque of a stimulated muscle [10]. This process corresponds to a certain approximation of the impulse response of a critically-damped second-order system in conjunction with a dead time. They could show that the maximal torque was reached after at least 100 ms . This delay and the pulse wave transit time are enough to ensure that the heart is in the diastolic filling phase.

**Figure 1:**
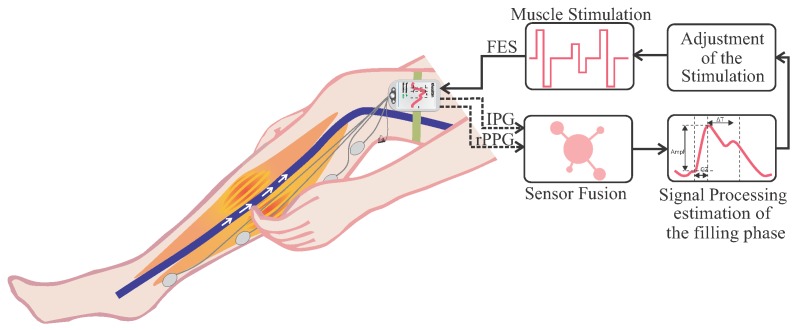
Schematic representation of the system. FES = functional electrical stimulation; IPG = impedance plethysmography; rPPG = reflective photoplethysmography

This adaptation of the stimulation has the advantage that the venous return arrives during the filling phase of the heart. In this way, heart valves are not harmed, and the heart is not unnecessarily burdened. Figure 1 presents a schematic representation of the system.

## 2. Sensor Device

**Figure 2:**
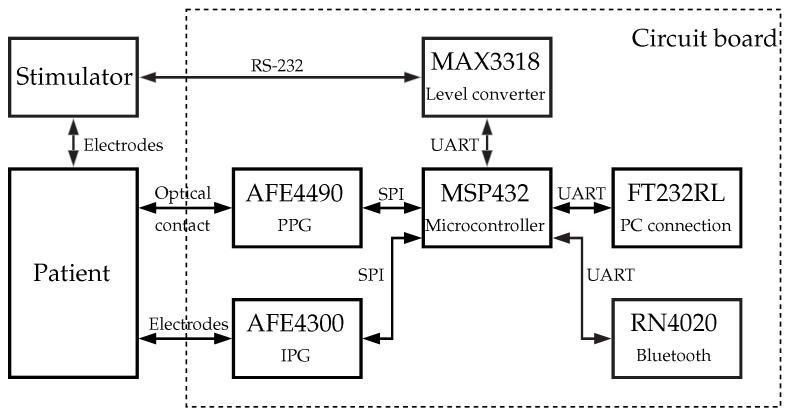
Schematic structure of the developed hardware.

The hardware design was developed as a double-sided PCB layout with dimensions of only 60 mm × 60 mm . Figure 2 presents a schematic diagram of the system.

The microcontroller MSP432P401R (Texas Instruments, Dallas, TX, USA) serves as the processing unit. The power supply of the system is realized via a USB connection or an attachable LiPo battery. The microcontroller works at a high clock frequency of 48 MHz to perform the necessary computational tasks. A delay between the raw data and the fusion signal should not be notable. Communication with peripheral devices (e.g., computers or smart phones) is possible via a USB connection or by Bluetooth.

For both IPG and PPG channels, integrated circuits were used (AFE4490 and AFE4300; Texas Instruments, Dallas, TX, USA). In addition to the hardware, the sensor data fusion plays an important role.

A state-space model was developed based on the principles of the Kalman filter [11]; this was necessary for the implementation into the microcontroller. The phase of the cardiac cycle is determined using the merged signal. This signal is then used for pulse-synchronized electro-stimulation of the muscle.

### 2.1. Impedance Plethysmography

Impedance plethysmography was firstly described by Nyboer [12] and aims at quantitatively determining blood volume changes of a tissue section. In IPG, a high frequency current (typically between 5 kHz and 100 kHz ) is injected into the tissue by two electrodes. Two other electrodes are used to measure the corresponding voltage drop, which depends on the tissue composition. The impedance equals the quotient of the measured voltage and injected current. The relationship between the measured impedance and changes of blood volume in the measured segment as described by Nyboer [12] is given by:

(1) $$\begin{array}{c} \frac{\Delta V}{V}=-k\;\cdot \;\frac{\Delta Z}{Z} \end{array}$$

ΔV is the blood volume change in the segment; *V* is the total volume of the examined tissue; *k* is a proportionality constant; ΔZ is the blood-related impedance change; and *Z* is the basal impedance. This constant *k* depends on two factors. First, it is determined by the composition of the examined body segment. Second, it depends on the measuring frequency. For example, a proportionality constant of k≈1 can be assumed for a measurement at the mid-calf and a frequency of 100 kHz [13]. The IPG was realized using the IC AFE4300 (Texas Instruments, Dallas, TX, USA). Measurements were made using commercial, adhesive ECG electrodes in a tetrapolar measurement setup. ECG electrodes are typically used for IPG applications, since they provide a low skin-electrode impedance. Details on the AFE4300 are available from the manufacturer’s data sheet [14].

Since the intended use of the AFE4300 is a body fat scale, injection and measurement of the current is performed between the surfaces of the two feet. In this way, the expected voltage drop is much higher than when used as an IPG system where only the lower leg is examined. Consequently, a lower impedance is measured. Furthermore, for pulse detection, a very small time-variant change must be detected.

**Figure 3:**
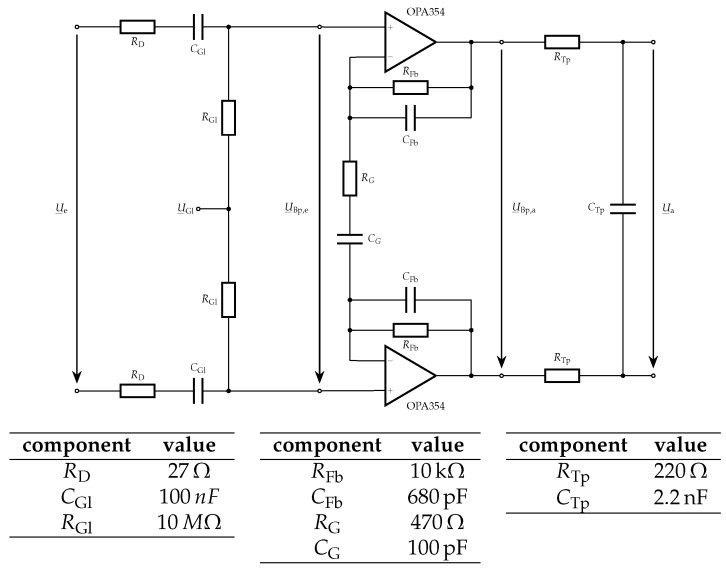
Schematic of the active bandpass filter to increase the relevant signal components.

The simplest way to use this integrated circuit as an IPG device is by amplification of the signal before it is passed to the AFE4300. Due to its differential inputs, a differential amplifier stage with a frequency-dependent characteristic curve was integrated in the measuring system (Figure 3).

**Figure 4:**
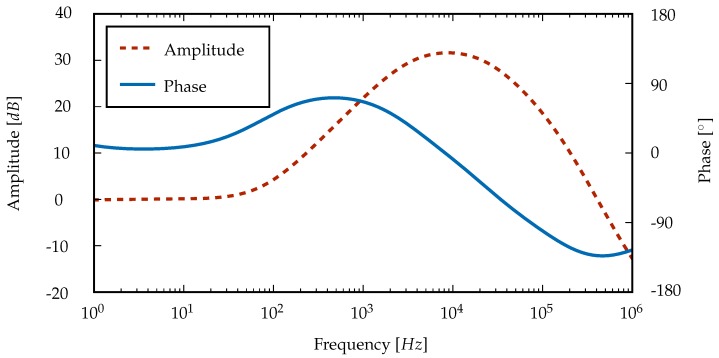
Amplitude and phase response of the active bandpass filter.

The circuit was simulated in “LTspice”(Linear Technology, Milpitas, CA, USA), and the result of this analysis is shown in Figure 4 as a Bode plot. In the range of typical IPG excitation frequencies around 10 kHz , a selective increase of the signal amplitude is visible, so that the resolution available for heart rate detection after sampling is substantially higher. The bandpass filter has a center frequency of approximately 9 kHz and a 3 dB bandwidth of 26 kHz .

### 2.2. Reflective Photoplethysmography

Photoplethysmography makes use of a light source that is irradiated into the skin. The transmitted light intensity (PPG, transmission mode, e.g., finger pulse oximeter) or reflected light intensity (rPPG) depends mostly on the dermal blood volume that changes with the heartbeat, breathing cycles, autonomic thermal regulation, and so on. Compared to impedance plethysmography, the measured blood volume variation depends mainly on the dermal blood circulation, which can vary due to physiological processes, for example due to ambient temperature [15]. The systolic amplitude of the PPG is an indicator of the pulsatile changes in blood volume caused by arterial blood flow around the measurement site [16,17]. Furthermore, the systolic amplitude has been related to stroke volume [18]. Due to the dependence on dermal blood circulation, PPG is not used for volume estimation, like IPG, but as a clinical standard to estimate arterial oxygen saturation values.

The rPPG was realized using the IC AFE4490 (Texas Instruments, Dallas, TX, USA). The chip has a built-in constant current source for two different LEDs and a transimpedance amplifier and features a high resolution analog-digital converter (ADC). Details on the AFE4490 are available in the manufacturer’s data sheet [19].

Two LEDs with a spectral emission of $\lambda_{\mathrm{red}}=660nm$ and $\lambda_{\mathrm{infrared}}=940nm$ were connected as the H-bridge. As the sensor system, the SFH7050 (OSRAM Opto Semiconductors, Regensburg, Germany) was used. This is a fully-integrated optoelectronic sensor, featuring three emitters and one detector, especially designed and optimized for reflective pulse oximetry.

### 2.3. Stimulation

The stimulation device was developed by “*tic Medizintechnik GmbH & Co. KG*” (Dorsten, Germany). Conventional stimulation electrodes are placed caudally and cranially at the gastrocnemius muscle. The device is connected by a serial connection and controlled by a communication protocol, which allows changing the stimulation, e.g., the beginning, the stimulation strength and frequency.

In this work, the peaks of the plethysmography signal (caused by the arterial inflow) are used to start the muscle stimulation. The beat-to-beat intervals of the arterial signal are estimated, and 56% of this time span is used as the duration of stimulation. Furthermore, model analysis has shown that blood flow is at its maximum at 56% of the duration of a heart beat. This analysis can be found in Weyer *et al*. [20]. Besides 56%, other ratios are tested for the flow enhancement, as well.

To both prove and evaluate the increase in blood flow, the popliteal vein at the knee is measured by duplex ultrasonography.

## 3. Signal Processing

For real-time measurement, an interrupt-based sample routine for the two chips has been implemented into the microcontroller. In addition to the sampling of the sensor channels, there are two additional tasks. First, when all necessary samples are measured, the channels are merged by a Kalman filter in order to improve signal quality. Second, the merged signal is used to perform a pulse wave detection.

Furthermore, the data are transferred to peripheral devices. Data exchange is done using a package structure, which consists of a start sequence, the type of packet, the data and its length and a final CRC-32 checksum. Although data exchange is done using double-buffered DMA, the microcontroller must also interpret the received packets.

### 3.1. Sensor Fusion

In this work, the implementation of the Kalman filter was based on the CMSIS DSP Library [21]. In addition to the computing operations for use of the Kalman filter, a system model is essential; this is described below.

For the application of the Kalman filter, knowledge is required of the system behavior in the form of the matrices $A_{k}$ , $B_{k}$ and $C_{k}$ , as well as of the noise processes in the form of the covariance matrices $Q_{k}$ and $R_{k}$ .

The system model of the Kalman filter for physiological signals was published by Foussier *et al.* [22]. In this work, the Kalman filter produces the system behavior of a simple sinusoidal oscillation with the frequency of the pulse. Since a constant frequency was assumed, no input signal influences the state of the system; thus, $B_{k}=0$ . The continuous-time pulse signal is according to the underlying model in the following form:

(2) $$f\left(t\right)=\cos\left(2\pi f_{\mathrm{HR}}\;t\right)=\cos\left(\omega_{\mathrm{HR}}\;t\right)$$

The state-space representation of this work is a mathematical model of differential equations of a system, in which one state variable is matched to f(t) . The Taylor expansion is calculated for a function f(x) at a point *a*:

(3) $$Tf\left(x;a\right)=\sum_{n=0}^{\infty }\frac{f^{\left(n\right)}\left(a\right)}{n!}{\left(x-a\right)}^{n}$$

In order to keep the computational complexity of Kalman filtering as low as possible, only a few state variables should be used. Hence, only the first-order Taylor polynomial was used:

(4) $$T_{1}f\left(x;a\right)=f\left(a\right)+f^{\prime }\left(a\right)\;\cdot \;\left(x-a\right)$$

Based on this linearization, the function value after a small time interval Δt and at the actual time *t* can be approximated as:

(5) $$f\left(t+\Delta t\right)\approx T_{1}f\left(t+\Delta t\;;\;t\right)=f\left(t\right)+f^{\prime }\left(t\right)\Delta t$$

If the time interval Δt is the inverse of the sampling rate $f_{s}=\frac{1}{\Delta t}$ and t=kΔt , then the conversion into a discrete time description is:

(6) $$f\left(\left(k+1\right)\Delta t\right)\approx f\left(k\Delta t\right)+f^{\prime }\left(k\Delta t\right)\Delta t\;\Rightarrow \;f_{k+1}\approx f_{k}+f_{k}^{\prime }\Delta t$$

While $f_{k}$ can be interpreted as a state variable that follows the desired waveform and for which the transition equation is known, the equation of the state variable $f_{k}^{\prime }$ must be determined. Similar to the first state variable, the linearization represents an acceptable approximation: $f_{k+1}^{\prime }\approx f_{k}^{\prime }+f_{k}^{\prime \prime }\Delta t$ .

Due to the properties of f(t) , the second derivative is again the function itself, with the exception of the pre-factors. The transition equation for the second condition variable is:

(7) $$\frac{d}{dt}f^{\prime }\left(t\right)=-\omega_{\mathrm{HR}}^{2}\;\cos\left(\omega_{\mathrm{HR}}t\right)=-\omega_{\mathrm{HR}}^{2}\;f\left(t\right)\;\Rightarrow \;f_{k+1}^{\prime }\approx f_{k}^{\prime }-\omega_{\mathrm{HR}}^{2}\;\Delta t\;f_{k}$$

The measured values of both plethysmography types are a superposition of small pulsatile components and large, slowly-varying proportions, which are partly influenced by venous blood. The output of the Kalman filter can represent both parts. Nonetheless, a simple linear combination of the two state variables $f_{k}$ and $f_{k}^{\prime }$ could not model the low-frequency components.

For this reason, additional state variables $g_{l,k}$ ( l=1,..,p ) must be introduced for each of the *p* sensor channels, which represent these slow proportions. The observation matrix $C_{k}$ can be chosen such that, for each sensor channel, the superposition of the two parts at the system output $y_{k}$ can be determined.

The transition equations for the slow part $g_{l,k}$ are formulated under the assumption of a constant value. Therefore, each part depends on the next time step from itself: $g_{l,k+1}=g_{l,k}$ .

Due to the low changing rate of the system, the estimated value could be adjusted to the measured value in each correction step.

The two PPG channels and the IPG channel result in the following transition equation. The slow changing parts $g_{1,k},\;g_{2,k}\;...$ are indexed as a type of plethysmography.

(8) $$\left(\;\underset{x_{k+1}}{\underset{︸}{\begin{array}{c} f_{k+1}\\ f_{k+1}^{\prime }\\ g_{{\mathrm{PPG}}_{\mathrm{IR}},k+1}\\ g_{{\mathrm{PPG}}_{\mathrm{RED}},k+1}\\ g_{\mathrm{IPG},k+1} \end{array}}}\;\right)=\left(\;\underset{A_{k}}{\underset{︸}{\begin{array}{ccccc} 1 & \Delta t & 0 & 0 & 0\\ -\omega_{\mathrm{HR}}^{2}\Delta t & 1 & 0 & 0 & 0\\ 0 & 0 & 1 & 0 & 0\\ 0 & 0 & 0 & 1 & 0\\ 0 & 0 & 0 & 0 & 1 \end{array}}}\;\right)\left(\;\underset{x_{k}}{\underset{︸}{\begin{array}{c} f_{k}\\ f_{k}^{\prime }\\ g_{{\mathrm{PPG}}_{\mathrm{IR}},k}\\ g_{{\mathrm{PPG}}_{\mathrm{RED}},k}\\ g_{\mathrm{IPG},k} \end{array}}}\;\right)+w_{k}$$

Since the measured values of the three plethysmography-channels are used as output variables of the system, the dimensions of the observation matrix $C_{K}$ are given as 3×5 . Each sensor channel is a linear combination of both state variables, the slowly varying proportion $g_{l,k}$ and the relevant component of the heart rate $f_{k}$ .

The pulse-synchronous part was weighted by a different scaling factor for each channel $h_{l,k}$ to enhance the signal quality.

(9) $$\left(\;\underset{y_{k}}{\underset{︸}{\begin{array}{c} y_{{\mathrm{PPG}}_{\mathrm{IR}},k}\\ y_{{\mathrm{PPG}}_{\mathrm{RED}},k}\\ y_{\mathrm{IPG},k} \end{array}}}\;\right)=\left(\;\underset{C_{k}}{\underset{︸}{\begin{array}{ccccc} h_{{\mathrm{PPG}}_{\mathrm{IR}},k} & 0 & 1 & 0 & 0\\ h_{{\mathrm{PPG}}_{\mathrm{RED}},k} & 0 & 0 & 1 & 0\\ h_{\mathrm{IPG},k} & 0 & 0 & 0 & 1 \end{array}}}\;\right)x_{k}\;+\;v_{k}$$

The remaining step to complete the system model is the appropriate choice of the covariance matrices of the system and measurement noise $Q_{k}$ and $R_{k}$ . For simplification, it is accepted that in each case, there is no correlation, so that the two matrices have only diagonal elements. The remaining elements are determined by trial and error, so that the result of filtering fits the expected values.

The choice of the starting values ${\hat{x}}_{0|0}$ and $P_{0|0}$ is not critical because an inappropriate choice only leads to a slower convergence velocity.

### 3.2. Pulse Detection

To perform the pulse-synchronized electro-stimulation, the phase of the cardiac cycle (estimated by the Kalman filter) has to be determined. The detection of pulse peaks from the plethysmography signals is difficult, not only because of the physiological variability of pulse peaks, but also due to the respiration, motion artifacts and electrical interference noise [23]. There are real-time heart rate detection algorithms for ECG or fingertip PPG [24,25,26,27]. However, the real-time detection of the heart rate from the merged IPG and rPPG signal (with limited computational capacity) measured at the leg is a new challenge. Since this is a real-time task, the algorithm operates in the time domain. More specifically, a relatively simple detection of local maxima was implemented for phase determination. The condition was defined such that the existence of a local maximum at the time *k* must apply:

(10) $$f_{k-1}\leq f_{k}\geq f_{k+1}$$

The signal $f_{k}$ is the merged signal of the Kalman filter. Since noise and physiological processes would produce several local maxima per heart beat, two other signal processing tools were used: a sliding averaging is used for smoothing the noise processes and an additional condition was used to avoid the detection of physiologically-related side maxima. Thus, the algorithm accepts the local maxima that satisfy the first condition, only at the beginning of a new pulse cycle when the following inequality is also satisfied:

(11) $$\begin{array}{cc} f_{k} & >\alpha \;\sigma_{f_{k|k-L}}+\mu_{f_{k|k-L}} \end{array}$$

$$\begin{array}{cc} \text{with:} & \\ \alpha & :\;\text{scaling}\;\text{factor}\;\text{for}\;\text{sensitivity}\;\text{adjustment}\\ \sigma_{f_{k|k-L}} & :\;\text{estimated}\;\text{standard}\;\text{deviation}\;\text{of}\;f\;\text{at}\;\text{the}\;\text{time}\;k\;\text{under}\;\text{consideration}\;\text{of}\;f_{k}\\ & \;\;\text{and}\;L\;\text{recent}\;\text{values}\\ \mu_{f_{k|k-L}} & :\;\text{estimated}\;\text{average}\;\text{of}\;f\;\text{at}\;\text{the}\;\text{time}\;k\;\text{considering}\;f_{k}\;\text{and}\;L\;\text{recent}\;\text{values} \end{array}$$

Calculation of the mean value and standard deviation was performed within a sliding window of *L* recent values. The estimated standard deviation was the square root of the corrected sample variance $\sigma_{f_{k|k-L}}^{2}$ . Thus, the two variables can be calculated as follows:

(12) $$\sigma_{f_{k|k-L}}=\sqrt{\sigma_{f_{k|k-L}}^{2}}=\sqrt{\frac{1}{L}\;\sum_{i=k-L}^{k}{\left(f_{i}-\mu_{f_{k|k-L}}\right)}^{2}}$$

(13) $$\mu_{f_{k|k-L}}=\frac{1}{L+1}\;\sum_{i=k-L}^{k}f_{i}$$

To keep the computation costs per iteration as low as possible, the calculations were implemented recursively, so that in each step, only the difference between the value at step *k* and k+1 was calculated.

(14) $$\begin{array}{c} \sigma_{f_{k+1|k-L+1}}^{2}-\sigma_{f_{k|k-L}}^{2}=\frac{1}{L}\left(\sum_{i=k-L+1}^{k+1}{\left(f_{i}-\mu_{f_{k+1|k-L+1}}\right)}^{2}-\sum_{i=k-L}^{k}{\left(f_{i}-\mu_{f_{k|k-L}}\right)}^{2}\right)\\ =\frac{1}{L}\left(\sum_{i=k-L+1}^{k+1}\left(f_{i}^{2}-2f_{i}\;\mu_{f_{k+1|k-L+1}}+\mu_{f_{k+1|k-L+1}}^{2}\right)-\sum_{i=k-L}^{k}\left(f_{i}^{2}-2f_{i}\;\mu_{f_{k|k-L}}+\mu_{f_{k|k-L}}^{2}\right)\right)\\ \begin{array}{cc} =\frac{1}{L} & (f_{k+1}^{2}+\left(L+1\right)\mu_{f_{k+1|k-L+1}}^{2}-2\mu_{f_{k+1|k-L+1}}\sum_{i=k-L+1}^{k+1}f_{i}\\ -\left(f_{k-L}^{2}+\left(L+1\right)\mu_{f_{k|k-L}}^{2}-2\mu_{f_{k|k-L}}\sum_{i=k-L}^{k}f_{i}\right)) \end{array}\\ \overset{\left(13\right)}{=}\frac{1}{L}\left(f_{k+1}^{2}-f_{k-L}^{2}-\left(L+1\right)\left(\mu_{f_{k+1|k-L+1}}^{2}-\mu_{f_{k|k-L}}^{2}\right)\right) \end{array}$$

(15) $$\mu_{f_{k+1|k-L+1}}-\mu_{f_{k|k-L}}=\frac{1}{L+1}\left(\sum_{i=k-L+1}^{k+1}f_{i}-\sum_{i=k-L}^{k}f_{i}\right)=\frac{1}{L+1}\left(f_{k+1}-f_{k-L}\right)$$

In this representation, the new mean value and the corrected sample variance were calculated from the actual time step and the previous steps, by an arithmetic operation dependent on the window length *L*. Before the recursion could be used, the two variables were calculated using an initial window. In this work, the algorithm according to Welford has been used, which is especially suitable due to its numerical stability [28].

## 4. Results

### 4.1. Detection of Heart Rate

**Figure 5:**
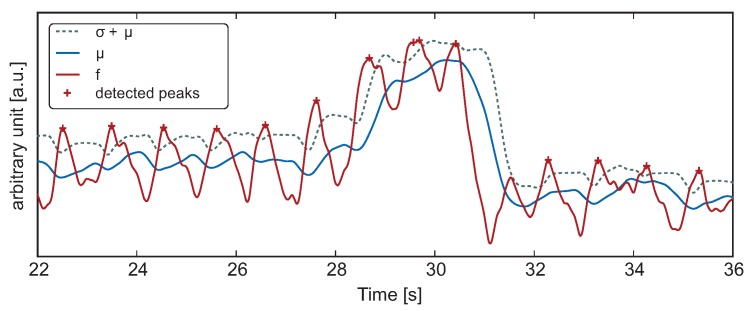
Pulse detection of a low quality signal ( L=49 , α=1 ). (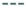) Sum of the standard deviation and the sliding average of the fused signal; (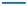) sliding average of the fused signal; (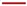) fused signal; (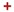) detected peaks in the fused signal.

Figure 5 shows a signal with an artifact. Most pulsations could be correctly identified by a suitable choice of the window length *L*. The slow adaptation of the detection interval is visible in the first six seconds. However, for the artifact at t≈29.5s , the algorithm is unable to prevent the false detection.

To analyze the performance of the system, a commercially available, transmissive PPG clip sensor (CMS50E, Contec Medical Systems, Qinhuangdao, China) was used as the gold standard. The sensor was fixed on the second toe. The rPPG was placed in the pit of the knee (at the back of the knee joint), and the electrodes of the IPG were placed on the rear side of the lower leg (just below the knee and slightly above the ankle).

In this experimental model, the Kalman filter and the parameters for the pulse detection were assumed to be as follows:

$f_{HR}=1\mathrm{Hz}=60\;\min^{-1};\;\;h_{l,k}=1\;\forall l;\;\;Q_{k}=0.001\;\cdot \;I_{5};\;\;R_{k}=I_{3};\;\;L=49;\;\;\alpha =0.95$

**Figure 6:**
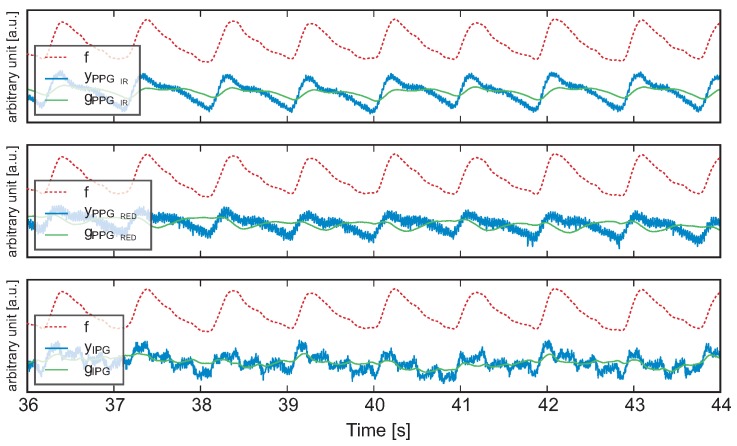
(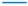) Raw data of each measurement channel $y_{l}$ , Kalman-filter: low frequency component $g_{l}$ of each measurement channel (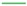) and the result of the fusion *f* (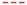).

**Figure 7:**
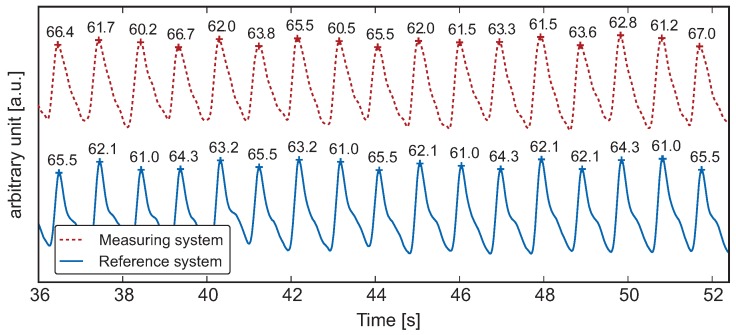
Comparison between measured values of the gold standard and the result of fusion *f* at good signal quality. All heart rates in beats per minute.

Figure 6 shows the raw data $y_{l}$ of all channels without further signal processing, as well as the low-frequency components $g_{l}$ and the merged pulse-synchronous component *f* from the Kalman filter. For a simple comparison with the gold standard, the signals were filtered, and peak detection was performed; the results are shown in Figure 7. Overall, a good correlation was observed between the two curves. Figure 7 also shows the heart rates (in $\min^{-1}$ ) calculated from the period between the two maxima. The averaging over several heartbeats was omitted since the device should produce meaningful values, even with irregular cardiac activity.

**Figure 8:**
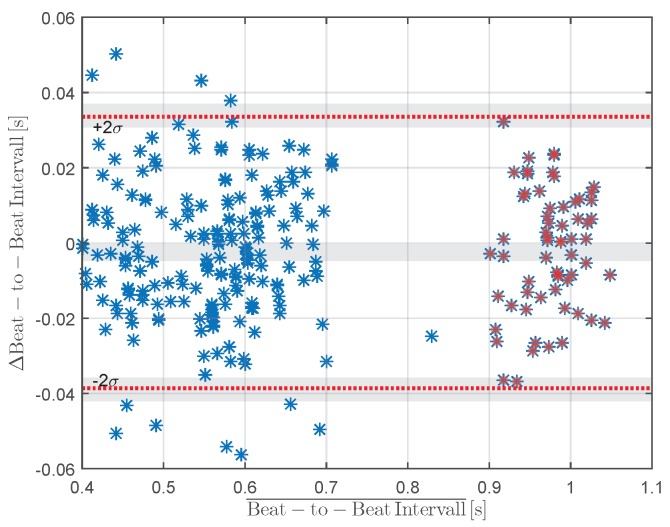
Bland–Altman diagram of the developed system and a commercial PPG finger clip as the gold standard at rest (red symbols) and after physical exertion (blue symbols).

During physical rest, all pulse peaks were correctly identified during a 60 s measurement period (sensitivity 100%, positive predictive value 100%). In a second experiment, the measurements were repeated after physical exertion. The measurement period of the second experiment was 120 s ; in this latter case, 202 correct peaks were found (true-positive); four were not detected (false-negative); and five additional wrong peaks were detected (false-positive). In this case, the algorithm detects a peak, which was caused by noise, and misdiagnosed it as heart related. The evaluation resulted in a sensitivity of 98% and a positive predictive value of 99%. For the evaluation of the accuracy of the beat-to-beat interval, a Bland–Altman plot was used to illustrate the performance (Figure 8) [29]. The horizontal lines are drawn at the mean difference and at the limits of agreement (at 0.033 s and -0.038 s ), defined as the mean difference plus and minus 1.96-times the standard deviation *σ* of the differences. Assuming a normal distribution, this interval indicates the area in which a share of 95% of the measured values can be expected.

The Bland–Altman diagram shows that there is no systematic error, because the differences are close to zero. The red symbols in Figure 8 indicate measurements during physical rest.

### 4.2. Flow Enhancement

Blood flow in the popliteal vein was observed with the help of ultrasound. The study was carried out on a healthy 30-year-old during a self-experiment. Due to the missing clinical approval of the device, it was only tested in a feasibility study on one subject in a self-experiment, and accordingly, the statistical significance is limited. The investigation was performed with regards to the local ethics committee. Although the stimulation intensity was set very low so that it was barely noticeable, it did cause a slight muscle twitch. The stimulations were done by a biphasic signal with an amplitude of 35 mA and a stimulation frequency of 50 Hz .

**Figure 9:**
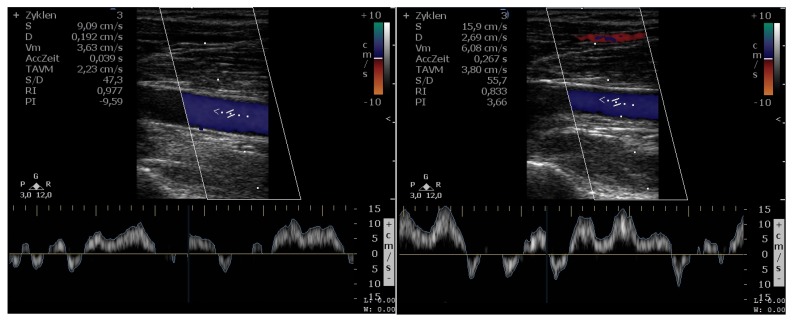
Duplex ultrasonography of the vein. Left: ratio of the stimulation duration to the beat-to-beat interval of 40%; right: ratio of the stimulation duration to the beat-to-beat interval of 56%.

Figure 9 exemplarily shows the results of the duplex ultrasonography, with a ratio of stimulation duration to beat-to-beat interval of 40% and 56%, respectively. The Doppler waveforms of the vein are shown in the lower part of the images. Furthermore, the automatic algorithm provided by the HD11 XE Ultrasound System (Philips) analyzed these waveforms and estimated the time-averaged mean velocity ( TAVM ) and the time-averaged maximum velocity ( Vm ) in the vein over three periods in $cm\;s^{-1}$ .

**Figure 10:**
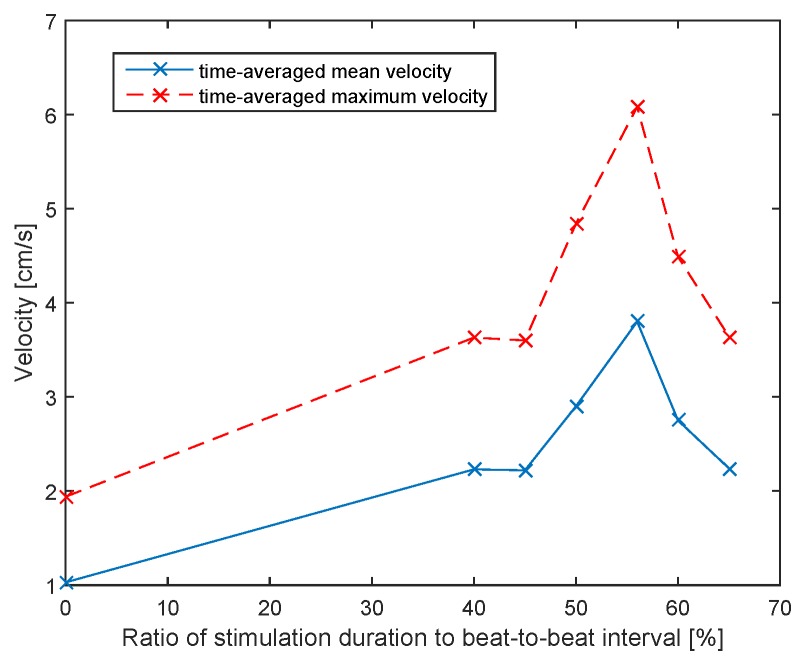
Time-averaged mean velocity and maximum velocity of the flow in the popliteal vein depending on the ratio of the stimulation duration to the beat-to-beat interval.

Figure 10 shows the Vm and TAVM of the flow in the popliteal vein depending on the ratio of the stimulation duration to the beat-to-beat interval. It was found that a maximum flow increase was reached at a ratio between 50% and 60%. This study demonstrates the feasibility of the system and the important impact of the adaptation of the stimulation to the heart rate, since higher flow rates can be achieved with adequate adaptation.

## 5. Discussion

The aim of this work was to develop the hardware and software for pulse detection on the human leg by means of two plethysmography channels. Using integrated circuits, a compact board was designed and built that fulfills all algorithmic requirements. A Kalman filter was designed for sensor data fusion. The device provides a reliable signal quality by merging reflective photoplethysmography and impedance plethysmography signal channels using a Kalman filter. On-line peak detection was used to determine the current phase of the heart rate from the merged signal.

The false-positive findings after physical exertion resulted from a difference between the heart rate in the system model of the Kalman filter and the actual heart rate. This problem can be solved by an adaptive adjustment of the Kalman filter, as proposed by Foussier *et al*. in [30]. In addition, a reduction of the error of the system model by a non-linear model would improve the system (extended Kalman filter). Furthermore, false detection can be avoided by a plausibility check, since rapid and large changes in heart rate are unusual. Nevertheless, a high sensitivity of approximately 98% was achieved. Furthermore, in addition to improvements in the software, a larger distance between the LED and photodiode might increase the penetration depth of the light and, thus, improve signal quality.

The ultrasound-based evaluation of the stimulation demonstrated the benefit of the adaptive stimulation. The results show highly increased blood flow in the popliteal vein at the knee. A more extensive study, with more subjects, is planned to investigate the influence of different stimulation parameters on blood flow.
